# Innovative models of care for LGBTQ+ older adults: a scoping review protocol

**DOI:** 10.1136/bmjopen-2025-115403

**Published:** 2026-04-27

**Authors:** Cameron Pattinson, P J Annand

**Affiliations:** 1 Centre for Care, Faculty of Social Sciences, The University of Sheffield Faculty of Social Sciences, Sheffield, UK; 2 Institute of Psychiatry Psychology and Neuroscience - Department of Health Service and Population Research, Service User Research Enterprise, King’s College London, London, UK

**Keywords:** Aging, Health Equity, Social Support, Sexual and Gender Minorities

## Abstract

**Abstract:**

**Objective:**

This study aims to identify and map innovative care models for LGBTQ+ older adults and characterise the supporting evidence base.

**Introduction:**

Older LGBTQ+ adults face persistent inequalities in health and social care, including discrimination, invisibility and barriers to appropriate support. As the population of openly LGBTQ+ older people grows, ensuring equitable and affirming provision has become increasingly urgent in the UK and beyond. In response, care models that may be considered innovative (whether newly developed, adapted for LGBTQ+ communities or applied in novel ways) have begun to emerge. However, no review has yet systematically mapped what is currently known about innovations in LGBTQ+ care and the evidence supporting them. This scoping review therefore aims to identify and map innovative care models for LGBTQ+ older adults and to characterise the supporting evidence base.

**Methods and analysis:**

This scoping review will be conducted in accordance with Arksey and O’Malley’s framework and reported using the Preferred Reporting Items for Systematic Reviews and Meta-Analyses Extension for Scoping Reviews (PRISMA-ScR) checklist. Evidence will be included where a care model or initiative is described or evaluated along with its implications for LGBTQ+ older people. For this review, care models are defined as organised approaches to care—that is, services, policies, practices (including training), initiatives or interventions—implemented in formal or informal care settings—such as residential care homes, housing with care schemes (eg, supported accommodation, retirement communities), domiciliary care, communities or informal long-term care arrangements. Use of the term ‘innovation’ will not be required for inclusion and will instead be coded by reviewers during charting. English-language sources of any design published between 2015 and 2025 will be included; sources without LGBTQ+ relevance, purely theoretical pieces, protocol papers and conference abstracts without full reports will be excluded. Searches will be conducted in Scopus, Social Science Premium Collection, CINAHL and PsycINFO, with grey literature identified through Overton. Additional searching of SCIE, Google Scholar, relevant third-sector resources and reference lists will be undertaken following source selection. After de-duplication, two reviewers will independently screen titles/abstracts and full texts, with disagreements resolved through discussion or consultation with a third reviewer. Data will be charted on author and year, country, source type, participants or source focus, intended beneficiary population, intervention/model/practice described, study design or document design, context/setting, methods, type of evidence, innovation basis, innovation level/domain, reviewer rationale for innovation coding, outcome or implementation indicators reported, main findings relevant to the review question and limitations of the study or evidence. Findings will be presented in tables and through narrative synthesis to map the models of care, their innovative features and the nature of the evidence base, and to identify implications for UK policy, practice and research.

**Ethics and dissemination:**

As this review will use data from publicly available sources, ethical approval is not required. Findings will be disseminated through peer-reviewed publication, presentations and accessible outputs for relevant stakeholders, including community-facing materials where appropriate.

**STRENGTHS AND LIMITATIONS OF THIS STUDY**

- This scoping review uses a broad search strategy across academic databases and grey literature sources to capture evidence from research, policy and practice.
- The review adopts a broad definition of ‘models of care’ and treats innovation as an analytical lens rather than an inclusion criterion.
- Inclusion of peer-reviewed and non-peer-reviewed sources enables identification of practice-based and policy-relevant material alongside academic studies.
- The review will not formally assess the quality or risk of bias of included sources and will be restricted to English-language sources.
- Evidence relating to the most marginalised and minoritised LGBTQ+ communities may be limited by gaps in the available literature.

## Introduction

 As the first generations of openly LGBTQ+ people enter later life, a growing body of research highlights the unique needs and vulnerabilities of this population. Older lesbian, gay, bisexual, queer and trans individuals often grew up in eras of criminalisation, stigma and medical pathologisation, and these lifelong experiences of minority stress and discrimination continue to impact their health and well-being in older age.^1^ Compared with their heterosexual and cisgender peers, LGBTQ+ seniors are far more likely to be single, to live alone and to have no children, resulting in diminished traditional support networks. For example, only about a quarter of older LGB people see biological family at least weekly, versus more than half of heterosexual people.^2^ Many rely instead on ‘families of choice’—friends and community—which may shrink over time and lack legal recognition in care settings. These factors leave older LGBTQ+ adults at heightened risk of social isolation, poorer physical and mental health and unmet care needs. Indeed, a systematic review finds inequities spanning physical health, mental health, social care, exposure to violence and loneliness in this group.^3^ Notably, rates of depression and anxiety are higher among older LGBTQ+ people than in the general older population, and many report feeling invisible or misunderstood by service providers. Such disparities are compounded by ageing itself, as cumulative disadvantages intersect with the challenges of growing older.^1 3^

At the same time, sexual orientation, gender identity and intersex status should not be conflated. These dimensions of identity are associated with overlapping but distinct experiences in later life: for example, older trans adults may face challenges linked to gender identity discrimination, barriers to accessing affirming services and (mis-)recognition within care systems,^4^ while emerging work suggests that older intersex people may have particular concerns about ageing and aged care, including lack of practitioner knowledge, repeated demands to explain their bodies and fears about how visibly different bodies may be treated in care settings.^5^ There is also evidence of important subgroup variation within sexual minority populations: older lesbian women have been shown to face distinctive issues around support, resilience, disclosure and invisibility,^6^ whereas ageing research has often been disproportionately shaped by evidence on gay men and has underrepresented older women, particularly lesbians and bisexual women.^6 7^ By contrast, literature specific to older bisexual men, non-binary people and asexual people remains comparatively sparse,^8^ although emerging work suggests that asexual people may face pathologisation and ageist assumptions in healthcare and social service settings.^9^

A consistent theme in both academic and community studies is that mainstream services often fail to meet the needs of LGBTQ+ elders. In Stonewall’s survey of over 1000 LGBTQ+ people aged 55 and up, three in five respondents were ‘not confident’ that social care or housing providers would be able to meet their needs. Nearly half said they would feel uncomfortable being ‘out’ to care home staff, and a third would be uncomfortable disclosing their sexual orientation or trans status to a housing provider, paid carer or healthcare staff.^2^ These findings speak to a fear of discrimination in later life services. Many older LGBTQ+ people worry that entering mainstream retirement homes or care facilities could force them ‘back into the closet’ or subject them to prejudice.^3^ As one advocate observed, there are ‘very genuine fears, particularly among older people, that if they ever become frail, the dignity and autonomy that they have built for themselves in their own life will not be recognised by the providers of formal care’ (House of Lords debate, col. 1021).^10^ For trans and gender-diverse older adults, these concerns may be intensified by cisnormative and binary approaches to gender in health and social care.^4^ Conversely, research also shows that when older LGBTQ+ people find affirming environments and peer support, it can significantly improve their quality of life—for example, participation in LGBTQ-focused social groups has been linked to reduced loneliness and better mental health.^3^

Academic literature underscores the urgency of addressing these issues. The Secure, Accessible, Friendly and Equal Housing study has shown that older LGBTQ+ people’s housing preferences centre on three key themes: safety, comfort and trust; community and connections; imagining the future.^3^ The research emphasises the importance of trust and social networks in later-life housing decisions, warning against reductive approaches that treat LGBTQ+ seniors as isolated individuals rather than as people embedded in communities of support. Almack and colleagues have explored how older LGBTQ+ people navigate ageing and end-of-life with ‘chosen families’, highlighting the need for services to recognise these relationships and not default to heteronormative and cisnormative assumptions about next-of-kin.^11^

This body of research finds that practice-oriented approaches to LGBT inclusivity—such as acknowledging same-sex partners or close friends in care planning—are crucial for maintaining the dignity of LGBTQ+ older people, yet such practices are still not routine. Meanwhile, a review of health and care outcomes reports that older LGBTQ+ people face persistent health inequalities (from higher rates of poor self-rated health to greater loneliness) and often encounter formal care environments that ‘severely compromise’ their identities and relationships built over a lifetime.^12^ This research highlights how LGBTQ+ seniors often conceal their identity in care settings due to anticipated bias, at significant cost to their mental health. One Age UK^13^ study participant, a professional working with LGBT+ older people, reported that: ‘People hide their magazines, they take their pictures off the wall, because they don’t know who’s going to turn up’. It goes on to discuss the fears LGBTQ+ people express around dementia care, around whether their identities will be neglected and about additional complexities associated with their condition: ‘An older transgender person experiencing cognitive decline, for example, may forget who they have come out to, or indeed that they have transitioned’. The literature also reveals socio-economic vulnerabilities, such as older LGBTQ+ adults being less likely to be homeowners (with implications for financial security in retirement) and in some cases having lower income or savings, partly a legacy of past discrimination in employment.^14^ Trans older adults in particular may face increased socio-economic disadvantage, including greater risk of poverty, housing precarity and homelessness.^15^ Furthermore, while intersex communities have their own distinct history of medicalisation, invisibility and rights claims, the literature on older intersex people—while sparse—suggests that they too may face later-life inequalities in healthcare, housing, social support, financial stability, disability and recognition within policy and practice, while remaining largely overlooked in ageing research and service development.^16^ The literature thus suggests that LGBTQ+ people entering older age often constitute an underserved group, with distinct needs in housing, health and social support that demand targeted attention.^1^

In policy circles, there is growing recognition of this reality. The UK Government’s 2018 nationwide LGBT Survey (with over 108 000 respondents) revealed that despite legal equality, many LGBTQ+ people, especially older cohorts, still feel unable to be themselves in various aspects of life. In response, the Government’s LGBT Action Plan (2018) pledged reforms to improve healthcare, social care and housing outcomes for LGBTQ+ citizens, including commitments to ensure people feel ‘safe in their own homes and communities’ and to embed LGBT needs in health and care services.^17^ However, specific issues of older LGBT people received limited direct focus in that Action Plan, despite the importance of supporting this population being emphasised in Parliament.^10^ Since then, advocacy groups have kept up the pressure. The Government Equalities Office and National Health Service bodies have appointed advisers on LGBTQ+ health inequalities and launched inclusion initiatives, but concrete changes at the local level are still emerging. Some local authorities and charities have begun their own research and pilots. For example, Stonewall’s earlier report on lesbian, gay and bisexual people in later life^2^ provided a foundational evidence base on older LGBT needs in the UK, and more recent work by the LGBT Foundation’s Pride in Ageing programme in Manchester has demonstrated the value of coproducing services with older LGBTQ+ people to reduce loneliness and improve confidence in accessing support.^18^ The Northern Health Science Alliance’s Ageing in the North report^19^ likewise highlights that addressing inequalities in ageing must include an LGBTQ+ lens, noting that older LGBT residents in northern England often report poorer health and difficulty accessing non-LGBT-specific services. There is also progress in the realm of housing with the UK’s first LGBTQ+ affirming retirement community, Tonic@Bankhouse in London, opening in 2021 and demonstrating a new model of inclusive ‘extra-care’ housing. All 19 flats in that scheme were quickly filled, underlining a ‘clearly defined need and demand’ for such provision.^20^ A similar ambition is now taking shape in Manchester, where plans have been approved for the country’s first purpose-built LGBTQ+ extra care residence of over 100 homes.^21 22^ These developments reflect growing momentum to ensure that older LGBTQ+ people can age with dignity, community and pride, rather than feeling forced into hiding or isolation in their later years.

### Rationale

The urgency and importance of research into and review of LGBTQ+ older people and care are evident. Older LGBTQ+ adults in the UK face well-documented disparities in health, housing and social support. They are more likely to be isolated, to lack family care and to feel uncomfortable or unsafe in mainstream care environments. These challenges carry real human costs—from mental health struggles to people going back ‘into the closet’ out of fear—and they demand a policy and practice response. This scoping review aims to synthesise existing research on new and evolving models of care for older LGBTQ+ people, the outcomes of these innovative models of care and the gaps in knowledge and understanding needed to best support this group.

A preliminary search of Scopus, the Cochrane Database of Systematic Reviews and JBI (Joanna Briggs Institute) Evidence Synthesis was conducted and no current or underway systematic reviews or scoping reviews on the topic were identified. Our study will review primary research from the academic and grey literature that focuses on LGBTQ+ older adults, where a care model or initiative in the UK or an international context is described or evaluated. For this review, care models are defined as organised approaches to care—that is, services, policies, practices, initiatives or interventions—implemented in formal or informal settings—such as residential care homes, housing with care schemes (eg, supported accommodation, retirement communities), domiciliary care, communities or informal long-term care settings. Use of the term ‘innovation’ is not required for inclusion; indicators of innovation will be captured at data charting. English-language sources of any design will be included; sources without LGBTQ+ relevance, purely theoretical pieces, protocol papers and conference abstracts without full reports will be excluded. Evidence will be limited to the past 10 years to help ensure results reflect contemporary and emerging models of care, in light of our focus on innovative practice.

### Review question

#### Primary research question

What models of care for older LGBTQ+ adults are described in the literature published between 2015–2025 across residential, housing-with-care and domiciliary and community/informal settings in the UK and internationally?

#### Subquestions

1. Models: Which models of care for LGBTQ+ older adults are described across the included literature, and in which settings are they located?
2. Innovation: Which identified models are described as innovative, or can be characterised as innovative on the basis of their features, and on what basis is that innovation understood?
3. Outcomes and implementation: What outcomes, implementation indicators, benefits, challenges and feasibility issues are reported in relation to these models?
4. Evidence gaps: What gaps exist in the evidence base, including gaps by setting, approach type, population and level of evaluation?

*Note:* ‘Innovation’ will be operationalised during data charting (eg, approaches categorised as novel/newly developed; adapted for LGBTQ+ older adults; first-in-setting/demonstrator; systematised/scaled quality; co-designed/participatory approach; tech-enabled adaptation) and treated as a secondary analytical lens, not an inclusion criterion.

## Methods

The proposed scoping review will be conducted between November 2025 and July 2026. In accordance with Arksey and O’Malley,^23^ it will use the Preferred Reporting Items for Systematic Reviews and Meta-Analyses (PRISMA) Extension for Scoping Reviews checklist for planning and reporting. The scoping review will follow this protocol, with the charting form being piloted and iteratively refined as familiarity with the evidence base develops, and with any changes documented in the final review.

‘Older adults’ may be defined variably across the literature, and LGBTQ+ ageing work often uses thresholds such as 50+ or 55+.^24^ This review will not impose a fixed age cut-off, in order to capture how older age is defined across the literature; however, this may limit comparability between sources and may obscure differences between people at different stages of later life. Evidence will be included where it relates to LGBTQ+ older adults, either as an overall population or through specific subgroups, including gay, lesbian, bisexual, transgender, queer, intersex, non-binary, asexual, pansexual, agender and other identities within the LGBTQ+ umbrella. The review will consider sources describing models of care in formal or informal settings, within the UK and internationally, where these are either designed specifically for LGBTQ+ older people or are mainstream models that address LGBTQ+ older adults’ care needs, for example, through targeted training or inclusive practice initiatives.

Eligible sources may include primary research on the description, development, implementation or evaluation of such models, as well as policy documents, reports and commentaries that directly describe existing models of care or interventions already in use. Sources without LGBTQ+ relevance, commentaries without direct reference to existing models or interventions, and sources limited to recommendations for future practice only will be excluded.

### Patient and public involvement

Members of the LGBTQ+ community, including people with relevant lived experience and/or practice expertise, will inform the review through advisory input. Their involvement is intended to support relevance, clarity and sensitivity in the interpretation of evidence and communication of results.

### Search strategy

The search strategy was developed with input from a librarian experienced in evidence retrieval. It aims to locate both peer-reviewed and unpublished sources, as well as grey literature such as policy documents. An initial limited search of Scopus will be conducted to identify articles on the topic, leading to identification of any overlooked potential keywords by examining the keywords associated with each abstract. The search string will be refined and developed for Scopus, and then adapted for The Social Sciences Premium Collection, CINAHL and PsycINFO (see online supplemental appendix #1 for the search strings for each database). A separate search string will be developed for Overton (see online supplemental appendix #1) as the original search string will be inappropriate for the database, as it cannot be limited by abstract and/or keywords. Each search will be limited to the past 10 years (2015–2025).

The searches will be conducted in Scopus, The Social Sciences Premium Collection, CINAHL, PsycINFO and Overton. The first four have been chosen to capture interdisciplinary literature across health, social care, psychology and social science, while Overton and Google Advanced Search will be used to identify policy and grey literature. The identified texts will go ahead for screening, and once they have been screened, key texts will be identified and entered into Connected Papers, to ensure there are no key texts missed. The reference lists of all key texts will also be examined to check for missing sources.

If there is no English-language translation available for texts, they will be excluded. Sources from 2015 to 2025 will be considered. If the university does not have access to texts, they will be requested from the library or authors of texts will be contacted.

### Source selection

Following the search, all identified citations and abstracts from Scopus, CINAHL, The Social Sciences Premium Collection, PsycINFO and Overton will be collated and uploaded into Covidence, which will automatically detect duplicate citations. Each duplicate will also be manually checked. Two reviewers will be assigned to the abstract screening stage for assessment against the inclusion criteria for the review. Potentially relevant texts will then be selected for a full text review.

The full text of selected citations will be assessed in detail against the inclusion criteria by two reviewers. Reasons for exclusion of sources of evidence at full text that do not meet the inclusion criteria will be recorded and reported in Covidence and exported later for the scoping review. Any disagreements that arise between the reviewers at each stage of the selection process will be resolved through discussion, or with an additional reviewer.

After the full texts have been reviewed, a list of five key texts will be identified and entered into Connected Papers to identify any missing texts from the review list. The reference lists of final texts will also be examined for any missing texts. If there are any, they will then go through the same abstract screening and full text screening process.

The results of the search and the study inclusion process will be reported in full in the final scoping review and presented in a PRISMA flow diagram.^25^

### Data extraction

Data will be charted from included sources of evidence using a draft charting form developed by the review team and piloted on a sample of included texts. The form will capture, where applicable: title, authors, year, country, source type, participants/source focus, intended beneficiary population (including relevant subgroup characteristics, such as sexual orientation, gender identity and intersex status), intervention/model/practice described, study design/document design, context/setting, innovation basis, innovation level/domain, reviewer rationale for innovation coding, outcome measures and/or implementation indicators, main findings relevant to the review question and limitations of study/evidence. Where reported, charting will also note whether findings relate to specific sexual orientation, gender identity or intersex subgroups, in order to identify subgroup-specific patterns and gaps in the evidence base. The charting form may be iteratively refined during the review process, with any amendments described in the final review. Data will be charted by two reviewers with disagreements resolved through discussion and, where necessary, involvement of a third reviewer.

A draft extraction table is provided (see online supplemental appendix #2). The draft data extraction table will be modified and revised as necessary during the process of extracting data from each included evidence source. Modifications will be detailed in the final scoping review output. The data will be extracted by two reviewers to ensure consistency. Any disagreements that arise between the reviewers will be resolved through discussion, or with an additional reviewer/s. If appropriate, authors of sources will be contacted to request missing or additional data, where required. Quality appraisal and risk of bias assessment will not be undertaken, as the purpose of this scoping review is to map the range, characteristics and nature of the available evidence rather than to assess effectiveness or make judgements about evidential strength. Sources will instead be classified descriptively according to source type, study/document design and type of evidence.

### Data analysis and presentation

Included sources will first be mapped descriptively by country, source type, study or document design, setting, intended beneficiary population, approach type and the basis on which innovation is identified. The findings will then be synthesised narratively under three broad themes: (1) types of intervention, model or practice described; (2) how innovation is conceptualised and operationalised; and (3) the contexts and settings in which these approaches are implemented. Within and across these themes, sources will be compared with examine who the approaches are intended for, what makes them innovative and what outcomes or implementation indicators are reported. Where possible, findings will also be synthesised in relation to specific sexual orientation, gender identity or intersex subgroups, including where evidence is limited or absent. In line with Arksey and O’Malley’s framework,^23^ results will be presented in tabular, graphical and narrative formats, as appropriate to the nature of the evidence; for example, charts may be used to show the frequency and distribution of identified models of care and tables to summarise how innovation is characterised across sources. As no formal appraisal of methodological quality will be undertaken, the review will not rank sources according to strength of evidence and conclusions will focus on the extent, nature and characteristics of the literature. Synthesis will be primarily deductive, informed by the review questions and charting framework, while remaining open to inductive insights that emerge during charting and synthesis.

### Ethics and dissemination

This scoping review is based solely on analysis of publicly available published and unpublished sources. The findings will be disseminated through publication in a peer-reviewed journal and through presentations to academic, policy, practice and community audiences. To support accessibility and wider reach beyond academic publication, the dissemination phase will also include the development of outputs such as infographics and Easy Read documents, where appropriate. These outputs will be intended to support engagement with a wide range of stakeholders, including researchers, practitioners, policymakers, third-sector organisations and LGBTQ+ communities.

## Supplementary material

10.1136/bmjopen-2025-115403 online supplemental file 1
