# Supplementary material for: Innovative models of care for LGBTQ+ older adults: a scoping review protocol
Source: BMJ Open. 2026 Apr 27;16(4):e115403. doi: 10.1136/bmjopen-2025-115403 (PMC13141158; doi:10.1136/bmjopen-2025-115403)

# Appendices

### Appendix I: Search strategy

The search strings for each database are below, including the Boolean operators and limitations applied to each database.

**Scopus**

(In title) LGBT* OR LGBTQ* OR LGB* OR GLB* OR queer OR gay OR lesbian OR bisexual OR non-binary OR transgender OR intersex OR asexual OR pansexual OR agender OR "gender minorit*" OR “sexual minorit*”

AND

(title abstract keywords) older OR ageing OR aging OR “old* age” OR elderly OR “later life” OR senior* OR pensioner* OR retiree*

AND

(title abstract keywords) “social care” OR “long-term care” OR “residential care” OR “community care” OR “community home*” OR “community housing” OR “nursing home*” OR “domiciliary care” OR “home care” OR “housing with care” OR “extra care housing” OR “sheltered housing” OR “retirement village*” OR “retirement home*” OR “retirement housing” OR “retirement communit*” OR “supported living” OR “assisted living” OR “care home*” OR “extra care accommodation” OR “care facilit*” OR “long term care” OR “housing and care” OR “sheltered accommodation” OR "retirement accommodation”

AND

(title abstract keywords) intervention* OR scheme* OR facilit* OR product OR service* OR group* OR program* OR programme* OR approach* OR model* OR device* OR app* OR platform* OR telecare OR telehealth OR "assistive technolog*" OR "smart home*" OR support* OR network* OR resource* OR initiative* OR training OR workshop* OR outreach OR advocacy OR policy OR framework OR strategy OR guideline* OR implementation OR practice* OR evaluation OR pilot OR trial OR innovation OR "good practice"

Limited by: 2015-2025

********************

**Social Science Premium Collection**

Criminology Collection, Education Collection, International Bibliography of the Social Sciences, Library & Information Science Collection, Linguistics Collection, Politics Collection, Social Science Database, Sociology Collection (including ASSIA & Social Services Abstracts).

(Document title) LGBT* OR LGBTQ* OR LGB* OR GLB* OR queer OR gay OR lesbian OR bisexual OR non-binary OR transgender OR intersex OR asexual OR pansexual OR agender OR "gender minorit*" OR “sexual minorit*”

AND

(abstract-summary) older OR ageing OR aging OR “old* age” OR elderly OR “later life” OR senior* OR pensioner* OR retiree*

AND

(abstract-summary) “social care” OR “long-term care” OR “residential care” OR “community care” OR “community home*” OR “community housing” OR “nursing home*” OR “domiciliary care” OR “home care” OR “housing with care” OR “extra care housing” OR “sheltered housing” OR “retirement village*” OR “retirement home*” OR “retirement housing” OR “retirement communit*” OR “supported living” OR “assisted living” OR “care home*” OR “extra care accommodation” OR “care facilit*” OR “long term care” OR “housing and care” OR “sheltered accommodation” OR "retirement accommodation”

AND

(abstract-summary) intervention* OR scheme* OR facilit* OR product* OR service* OR group* OR program* OR programme* OR approach* OR model* OR device* OR app* OR platform* OR telecare OR telehealth OR "assistive technolog*" OR "smart home*" OR support* OR network* OR resource* OR initiative* OR training OR workshop* OR outreach OR advocacy OR policy OR framework OR strategy OR guideline* OR implementation OR practice* OR evaluation OR pilot OR trial OR innovation OR "good practice"

Limited by: 2015-2025

********************

**CINAHL**

(Document title) LGBT* OR LGBTQ* OR LGB* OR GLB* OR queer OR gay OR lesbian OR bisexual OR non-binary OR transgender OR intersex OR asexual OR pansexual OR agender OR "gender minorit*" OR “sexual minorit*”

AND

(Title and abstract) older OR ageing OR aging OR “old* age” OR elderly OR “later life” OR senior OR pensioner OR retiree*

AND

(Title and abstract) “social care” OR “long-term care” OR “residential care” OR “community care” OR “community home*” OR “community housing” OR “nursing home*” OR “domiciliary care” OR “home care” OR “housing with care” OR “extra care housing” OR “sheltered housing” OR “retirement village*” OR “retirement home*” OR “retirement housing” OR “retirement communit*” OR “supported living” OR “assisted living” OR “care home*” OR “extra care accommodation” OR “care facilit*” OR “long term care”OR “housing and care” OR “sheltered accommodation” OR "retirement accommodation”

AND

(Title and abstract) intervention* OR scheme* OR facilit* OR product* OR service* OR group* OR program* OR programme* OR approach* OR model* OR device* OR app* OR platform* OR telecare OR telehealth OR "assistive technolog*" OR "smart home*" OR support* OR network* OR resource* OR initiative* OR training OR workshop* OR outreach OR advocacy OR policy OR framework OR strategy OR guideline* OR implementation OR practice* OR evaluation OR pilot OR trial OR innovation OR "good practice"

Limited by: 2015-2025

********************

**PsycInfo**

(Title) LGBT* OR LGBTQ* OR LGB* OR GLB OR queer OR gay OR lesbian OR bisexual OR non-binary OR transgender OR intersex OR asexual OR pansexual OR agender OR “gender minorit*” OR “sexual minorit*”

AND

(Abstract) older OR ageing OR aging OR “old* age” OR elderly OR “later life” OR senior* OR pensioner* OR retiree*

AND

(Abstract) “social care” OR “long-term care” OR “residential care” OR “community care” OR “community home*” OR “community housing” OR “nursing home*” OR “domiciliary care” OR “home care” OR “housing with care” OR “extra care housing” OR “sheltered housing” OR “retirement village*” OR “retirement home*” OR “retirement housing” OR “retirement communit*” OR “supported living” OR “assisted living” OR “care home*” OR “extra care accommodation” OR “care facilit*” OR “long term care”OR “housing and care” OR “sheltered accommodation” OR "retirement accommodation”

AND

(Abstract) intervention* OR scheme* OR facilit* OR product* OR service* OR group* OR program* OR programme* OR approach* OR model* OR device* OR app* OR platform* OR telecare OR telehealth OR "assistive technolog*" OR "smart home*" OR support* OR network* OR resource* OR initiative* OR training OR workshop* OR outreach OR advocacy OR policy OR framework OR strategy OR guideline* OR implementation OR practice* OR evaluation OR pilot OR trial OR innovation OR "good practice"

Limited by: 2015-2025

********************

**Overton**

Title: LGB* AND “social care”

Title: LGB* AND ageing’

Title: LGB* AND older

Title: LGB* AND housing

Title: LGB* AND care AND older

********************

### Appendix II: Data extraction instrument


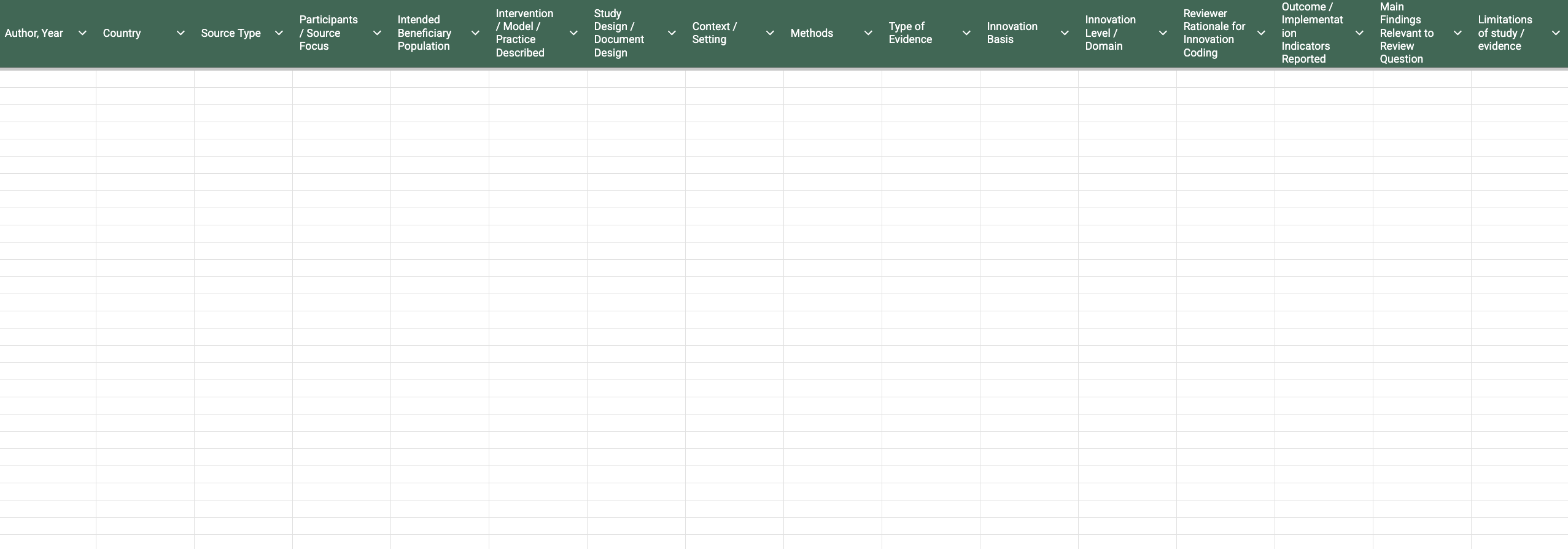

Supplement: online supplemental file 1 [file bmjopen-16-4-s001.docx]
