# Supplementary material for: Innovative models of care for LGBTQ+ older adults: a scoping review protocol
Source: BMJ Open. 2026 Apr 27;16(4):e115403. doi: 10.1136/bmjopen-2025-115403 (PMC13141158; doi:10.1136/bmjopen-2025-115403)
Supplement: Supplementary file 2 [file bmjopen-2025-115403.reviewer_comments.pdf]

PEER REVIEW HISTORY

BMJ Open publishes all reviews undertaken for accepted manuscripts. Reviewers are asked to complete a checklist review form and are provided with free text boxes to elaborate on their assessment. These free text comments are reproduced below.

ARTICLE DETAILS

Title (Provisional)

Innovative Models of Care for LGBTQ+ Older Adults: A Scoping Review Protocol

Authors

Pattinson, Cameron; Annand, PJ

VERSION 1 - REVIEW

**Reviewer**	**1**
**Name**	**Higgins , Agnes**
**Affiliation**	**School of Nursing and Midwifery, Trinity College Dublin**
**Date**	**02-Mar-2026**
**COI**	**None**

Thank you for asking me to review this paper. This is a welcome and important review. The following areas need further consideration.

The introduction section requires greater attention to the difference between gender and sexual orientation. Currently there appears to be a conflation of gender and sexual orientation issues with a grouping of LGBT identities. Mentions heterosexual peers (line 11) and heteronormative assumptions (line 58), however there is a need to explore how cisnormativity and a binary approach to gender impact trans and gender diverse older people.

Would like to see a greater exploration on the challenges transgender older people face, especially in context of housing and housing precarity- it is more than a fear of being out or forgetting about transitioned. The body of literature in relation to housing/homelessness will help strengthen the background and the rational for the review.

States that it will not formally assess the quality or risk of bias within the studies. In light of this comment, I am unclear how the level of evidence will be reported. As quality is a marker of the level of evidence, please explain the rationale for not assessing quality and how that impact on the reporting of level of evidence.

In the strengths and limitations section and in the search terms there is a mention and inclusion of Intersex, yet there is no mention of this group in any part of background. This needs to be addressed.

In data extraction, please explain if the key findings will be reported based on gender identity and sexual orientation. At the moment it is unclear. Suggest that findings are also narratively synthesised to bring to the fore any specific findings that relates to sender and sexual subgroups.

Older is defined as 55 plus, need a greater explanation as to why that is the age selected, plus the limitations of selecting that age.

Given the policy drive for PPI, will people form the LGBTQI+ community be involved in the review.

Please clarify if an information retrieval expert has been consulted regarding search strategy and search terms.

VERSION 1 - AUTHOR RESPONSE

<u>**Reviewer comments**</u>

1. The introduction section requires greater attention to the difference between gender and sexual orientation. Currently there appears to be a conflation of gender and sexual orientation issues with a grouping of LGBT identities. Mentions heterosexual peers and heteronormative assumptions, however there is a need to explore how cisnormativity and a binary approach to gender impact trans and gender diverse older people.

Thank you for this comment. Mentions of heteronormativity now explicitly also reference cisnormativity also (Introduction, paragraphs 1, 3 and 4).

2. Would like to see a greater exploration on the challenges transgender older people face, especially in context of housing and housing precarity - it is more than a fear of being out or forgetting about transitioned. The body of literature in relation to housing/homelessness will help strengthen the background and the rationale for the review.

We have strengthened the background section by expanding discussion of the literature on trans and gender diverse older people's experiences of discrimination, misrecognition, barriers to accessing affirming services (Introduction, paragraph 2), and housing inequality (Introduction, paragraph 5)

3. States that it will not formally assess the quality or risk of bias within the studies. In light of this comment, I am unclear how the level of evidence will be reported. As quality is a marker of the level of evidence, please explain the rationale for not assessing quality and how that impacts on the reporting of level of evidence.

We have clarified that *type* of evidence will be reported (rather than 'level'), as the purpose of the scoping review is to map the extent, range and characteristics of the

evidence rather than to determine intervention effectiveness or produce practice recommendations based on study quality, rather than presenting a formal hierarchy or graded assessment of the level of evidence. Instead, we will report the characteristics of the included evidence descriptively, including study design, methodology, population, setting, and publication type, so that readers can interpret the nature and breadth of the evidence base. (Abstract, Methods>Data extraction section, paragraph 2; Methods>Data analysis and presentation section, paragraph 1; Strengths and Limitations section, bullet 4)

4. In the strengths and limitations section and in the search terms there is a mention and inclusion of Intersex, yet there is no mention of this group in any part of background. This needs to be addressed.
Thank you for highlighting this. We have revised the background section to explicitly acknowledge intersex people within the wider field of LGBTQ+ ageing and housing research, also recognising that intersex experiences and needs may differ in important ways from those relating to sexual orientation and gender identity. (Introduction, paragraphs 2 and 5)

5. In data extraction, please explain if the key findings will be reported based on gender identity and sexual orientation. At the moment it is unclear. Suggest that findings are also narratively synthesised to bring to the fore any specific findings that relates to gender and sexual subgroups.
Thank you for this. We have clarified that, where reported in the included studies, findings will be extracted and presented by relevant subgroup characteristics, including sexual orientation, gender identity, and intersex status. (Methods>Data Extraction, Paragraph 1).

6. Older is defined as 55 plus, need a greater explanation as to why that is the age selected, plus the limitations of selecting that age.
The revised text now explains that definitions of older age vary across the literature, and that LGBTQ+ ageing research and policy often use thresholds such as 50+ or 55+, reflecting the cumulative effects of minority stress, earlier onset of some health and social inequalities, and the fact that LGBTQ+ communities and services may define "older age" differently from the general population. We have also added explicit discussion of the limitations of this threshold, including that it groups together individuals at quite different life stages and may obscure differences between those in their fifties and those in later life. (Methods section, paragraph 2)

7. Given the policy drive for PPI, will people from the LGBTQI+ community be involved in the review.
We have added a statement describing the involvement of people with relevant lived and professional experience in the review. (Methods section, paragraph 4)

8. Please clarify if an information retrieval expert has been consulted regarding search strategy and search terms.
We have added a sentence explaining that a librarian was consulted during the

development of the search strategy. (Search strategy section, paragraph 1)
